# Cerebral Small Vessel Disease in Population-Based Research: *What are We Looking at - and What not?*

**DOI:** 10.14336/AD.2023.0323

**Published:** 2024-08-01

**Authors:** Sanne S Mooldijk, M. Arfan Ikram

**Affiliations:** Department of Epidemiology, Erasmus MC University Medical Center, Rotterdam, the Netherlands

**Keywords:** cerebral small vessel disease, dementia, magnetic resonance imaging, population-based

## Abstract

Cerebral small vessel disease (CSVD) is considered as one of the main causes of cognitive decline and dementia. However, despite extensive research, the pathogenesis of CSVD and the mechanisms through which CSVD leads to its clinical manifestations remain largely unclear. The challenging in vivo quantification of CSVD hampers progress in further unraveling the pathogenesis and pathophysiology of CSVD. Currently, markers of CSVD are mainly brain abnormalities attributed to CSVD, but these are limited in reflecting morphological and functional changes of the microvasculature. We describe aspects of CSVD that are reflected by currently used techniques and those that are still insufficiently captured.

## Introduction

Dementia is one of the most common and devastating diseases in societies worldwide. Vascular risk factors are established drivers of several types of dementia [1]. Apart from pathology related to the large cerebral vessels, e.g., stroke, as an explanation for this link, the contribution of pathologies of the smaller brain vessels to cognitive decline and dementia is also acknowledged [2-4]. These pathologies, referred to as cerebral small vessel disease (CSVD), affect small arteries, arterioles, capillaries and venules. Pathological mechanisms underlying CSVD include hypoperfusion, inflammation, oxidative stress and disruption of the blood brain barrier resulting from systemic vascular and metabolic diseases [5]. However, large parts of the mechanisms contributing to CSVD as well its consequences remain poorly understood. It is therefore unsurprising that there are currently no specific treatment options. Progress in unraveling the pathogenesis of CSVD and the mechanisms linking CSVD to its clinical sequelae, such as cognitive decline and dementia, is hampered by the challenging quantification of CSVD. The cerebral small vessels themselves are not easily visualized in vivo, let alone their functioning. Currently used methods largely rely on brain imaging techniques that do not visualize the small vessels per se, but instead the damage to brain tissue which is thought to result from CSVD, such as white matter hyperintensities (WMH), lacunes and microbleeds [6]. These tissue pathologies have been adopted as markers of CSVD and are widely used in both research and clinical settings. Importantly, magnetic resonance imaging (MRI) based assessment of CSVD in large population-based cohort studies has provided valuable insights in the pathophysiology of CSVD and its role in dementia [7, 8]. Yet, these markers do not fully reflect small vessel integrity and functioning of the neurovascular unit. Particularly, they fail to capture the dynamic and heterogeneous nature of CSVD and, possibly for that reason, do not describe the wide variation in clinical manifestations. Here, we provide a brief overview of the presumed pathophysiology of CSVD, its suggested role in cognition declines and dementia, and presently used markers thereof. Finally, we describe shortcomings of those markers in reflecting CSVD, and we explain what aspects of CSVD require better markers.

## Small vessels in the brain: normal morphology and functioning

Cerebral small vessels include small arteries, arterioles, venules an capillaries, with diameters ranging from 5 μm to 2 mm [9]. Their main functions are to provide brain tissue with the oxygen and nutrients needed, to clear waste products from the brain and to form a barrier between the blood and brain (blood-brain barrier) which maintains the interstitial milieu of the brain [10]. For optimal metabolite exchange that matches with variations in metabolic demands, meticulous regulation of the blood flow in the vessel bed is essential. With neuronal activity, the metabolic demands will increase and the perfusion of the vessel bed will adapt correspondingly, a process called “neurovascular coupling” [11]. This process relies on the vasoreactivity of vessels: the ability of arterioles and capillaries to dilate in response to increased neuronal activity or a metabolic or vasodilatory stimulus. On a cellular level, interactions between endothelial cells, neurons, astrocytes, microglia, oligodendrocytes, and pericytes (in capillaries) or smooth muscle cells (in arterioles) are responsible for the proper functioning of this system [12, 13]. Together, these cells form the neurovascular unit. At the level of the small arteries and arterioles, smooth muscle cells adjust the vascular tone to match the regional metabolic needs of the downstream brain tissue. Similar functions are fulfilled by pericytes at the capillary level [14], in reaction to neuronal feedback, and chemical signals or mechanical forces on the endothelial cells [15, 16].

Unique in the brain vasculature is the blood-brain barrier which has a major role in the maintenance of the interstitial milieu. Junctional complexes between endothelial cells prevent bidirectional exchange of hydrophilic substances. Specialized transport proteins (GLUT1, LRP-1 etc.) are responsible for transporting nutrients from blood to brain and metabolic by-products from brain to blood. The blood-brain barrier is further composed by the basement membranes, pericytes and astrocyte end-feet [17].

## CSVD pathophysiology and pathology

CSVD denotes a range of pathological processes that affect the small arteries, arterioles, capillaries and venules and thereby lead to disturbances in the functions described above [5]. A small proportion of patients with CSVD suffers from hereditary disorders such as cerebral autosomal dominant arteriopathy with subcortical infarcts and leukoencephalopathy (CADASIL) and hereditary cerebral amyloid angiopathy (CAA), or from inflammatory and immunologically mediated small vessel diseases (e.g. vasculitis) [5]. More common is sporadic CSVD, for which two main types are recognized: hypertensive CSVD (also known as non-amyloid microangiopathy) and sporadic CAA (amyloid microangiopathy). In contrast to CSVD due to hereditary disorders, sporadic CSVD likely does not relate to one specific underlying disease, but is rather though to develop with accumulation of vascular risk factors, such as diabetes, hyperlipidemia, smoking, and in particular hypertension [18]. It should be noted that the distinction by etiology (i.e. hypertensive or amyloid) can be somewhat deceiving since it gives the impression that no other etiological factors were involved, while sporadic CSVD is considered a multifactorial disease. Moreover, the two types of microangiopathies may co-exist [9, 19].

### Pathogenesis and pathophysiology

Sporadic CSVD is thought to originate, at least for a substantial part, from known cardiovascular risk factors [20]. Through metabolic disturbances, including oxidative stress, inflammation, and hyperglycemia, and changes in cerebral blood flow, such as increased pulsatility, these cardiovascular risk factors may ultimately affect the small vessels in the brain. In the subsequent pathogenesis of CSVD, there are central roles for disturbances in blood flow-metabolism coupling, impaired vasoreactivity, narrowing of the vessel lumen, blood-brain barrier breakdown, ischemia, oxidative stress, inflammation and impaired interstitial fluid drainage [10, 21]. While these processes likely induce each other, it is yet to be determined which are the main initiators, and which are merely consequences that maintain the vicious cycle. Hypoxia, for instance, resulting from failing flow-metabolism coupling or inefficient oxygen extraction from the circulation, is known to damage endothelial cells and thereby the blood brain barrier [22]. The resulting leakage of fluids and toxic plasma components into the brain may consequently lead to increasing interstitial fluid and thickening and stiffening of vessel walls, further impairing the capacity of vasodilatation [10]. The pathological processes related to CSVD may induce brain tissue damage, such as neuronal injury or demyelination, which could ultimately be irreversible, e.g. neuronal death.

Another common pathology observed in CSVD is CAA, characterized by amyloid-β proteins in the tunica media and adventitia of small arteries and arterioles. CAA leads to degeneration of smooth muscle cells and pericytes, which may reduce vasodilatory abilities and increase the propensity for vessel rupture [23]. Simultaneously, existing small vessel dysfunctioning and related impairments of the lymphatic system, may reduce the elimination of amyloid-β from the brain and thus increase its deposition, from vessel walls or brain tissue [19]. Indeed, CSVD is thought to also aggravate parenchymal amyloid-β depositions, and vice versa. Especially in older adults, CSVD and other dementia-related pathologies, including amyloid-β, tauopathy, and synucleinopathies, often co-occur and are thought to interact [24-26].

### Histological abnormalities in CSVD

Several vascular abnormalities in the context of CSVD have been described in histological studies. These abnormalities include thickening of the vessel wall and of the basement membrane, vessel ruptures (possibly with micro aneurysms), narrowing of the lumen, enlarged perivascular spaces, depositions of hyaline substance in the vascular wall (lipohyalinosis), amyloid depositions in the vessel wall, loss of smooth muscle cells and depositions of collagen [27]. In late stages of the disease, vessels may be tortuous, or the total integrity of the vascular wall may be lost (fibrinoid necrosis) [23]. Nonfunctional capillaries with only a basement membrane and no endothelial cells can also be observed: “string vessels”.

## CSVD in cognitive impairment and dementia

CSVD is considered as one of the major causes of cognitive impairment and dementia and may be a link between systemic vascular risk factors and brain functioning [18]. Recently, CSVD was estimated to cause about 50% of dementias, not limited to dementia of the vascular type [10]. Indeed, post-mortem studies indicate that cerebrovascular pathologies are present in the majority of dementia patients and that they are more prevalent than in controls [28-30]. Longitudinal population-based studies have also shown associations of neuroimaging-based markers of CSVD with the risk of dementia [7].

The mechanistic basis of CSVD as a cause of dementia likely involves a reduced energy supply and impaired ability to clear accumulated waste, ultimately leading to parenchymal damage and neurodegeneration. Other brain pathologies such as protein depositions may aggravate, or perhaps induce, these processes. Dysregulation of cerebral blood flow, impaired vascular reactivity, and breakdown of the blood-brain barrier have been demonstrated to occur early in the development of dementia [12, 31]. Nevertheless, large parts of the pathway from vascular risk factors to dementia, mediated by CSVD, remain poorly understood.

## Visualization of CSVD in population-based research

For the current understanding of the pathophysiology of CSVD and the relation with cognitive impairment, experimental models and post-mortem studies with direct visualization of cerebral small vessels have made important contributions [5, 32, 33]. In addition to those studies, in vivo human studies are essential to unravel disease mechanisms and to bridge the gap between research and clinical practice. To that end, in vivo visualization of CSVD is implemented in clinical research settings, and increasingly in population-based cohorts as well, also referred to as epidemiological studies. The latter group of studies has certain advantages. First, they typically include participants years before the clinical symptoms of a disease can be observed. Owing to the longitudinal designs, the disease course including the preclinical phase is subsequently monitored. Thus, they are particularly suitable to study common diseases in the ageing population, such as dementia, with complex, multifactorial and interacting etiologies. Risk factors, pathologies and clinical presentations can be studied in their temporal order, which is essential to understand disease mechanisms. Assessment of potential risk factors before clinical symptoms manifest, or even before onset of pathologies, allows to determine directionality of effects.

Second, in more selected study populations (i.e., not community-based), different risk factors, pathologies and clinical presentations could be found, with possibly also different associations between them. Research in relatively unselected populations ensures that results apply to a broader underlying population.

With those advantages of population-based studies also come limitations. A relevant limitation in the context of CSVD is that certain techniques are less suitable in population-based studies. Especially more invasive measurements, for example imaging with contrast, are generally avoided to minimize the burden on the “healthy” participants. In addition, the size of these studies hampers implementation of more advanced, but expensive, techniques. Several techniques that are presently used are mentioned below. The Table 1 summarizes the value of each method as a marker of CSVD and lists their advantages and limitations.

### Magnetic resonance imaging

Brain MRI is the most widely used technique to assess CSVD in vivo. With conventional MRI, visible manifestations of CSVD include WMHs, lacunes (small fluid-filled cavities), microbleeds (small brain hemorrhages), cortical siderosis and enlarged perivascular spaces [6]. Importantly, these markers do not visualize the small vessels themselves. Instead, they are mostly parenchymal abnormalities that are assumed to result from impaired functionality of the cerebral small vessels, while microbleeds may come closer to reflecting small vessel integrity. Microinfarcts are another manifestation of CSVD that may be observed MRI with high resolution MRI (≥3 Tesla). For examination of the microstructural integrity of the white matter and changes therein, structural MRI can be complemented by diffusion-tensor imaging (DTI). These MRI techniques are noninvasive and well-defined criteria exist for the assessment for the CSVD related lesions [6]. As such, they are valuable in both clinical and research settings to detect brain damage attributed to CSVD. More advanced methods are also available, for example to further quantify blood-brain barrier permeability or the mismatch between capillary flow and oxygen delivery (capillary transit time heterogeneity), but these are more invasive as they rely on contrast agents and are thus not as widely used [21, 34].

**Table 1:** Overview of current techniques for in vivo assessment of cerebral small vessel disease.

Technique	Aspect of CSVD quantified	Advantages	Limitations
**MRI**			
**Conventional MRI (≤3 Tesla)** **(including T1-, T2-/FLAIR, PD-, and susceptibility weighted sequences) [6]**	Parenchymal abnormalities (white matter hyperintensities, lacunes, microbleeds, cortical siderosis, enlarged perivascular spaces)	Noninvasive; well-defined criteria	Parenchymal abnormalities visualized and not the small vessels per se
**Diffusion weighted imaging [DWI] (e.g., by diffusion tensor imaging [DTI]) [61]**	White matter integrity	Noninvasive; marker of white matter functionality	Reflects consequences of CSVD
**Ultrahigh resolution MRI (≥7 Tesla) [54]**	Smaller parenchymal abnormalities and smaller perforating arteries, e.g., micro-infarcts	Noninvasive; increased resolution	Costly; long scan times
**Contrast-based perfusion MRI** **(dynamic susceptibility contrast [DSC] MRI, dynamic contrast enhancement [DCE] MRI) [52, 56]**	Brain hemodynamics, cerebrovascular reactivity, and blood-brain barrier function (e.g. mean transit time, capillary transit time heterogeneity)	Quantification of brain hemodynamics including capillary flow, cerebrovascular reactivity, and blood-brain barrier function	Invasive
**Arterial spin labelling perfusion MRI [55]**	Blood-brain barrier permeability and capillary flow	Noninvasive; metrics of blood-brain barrier permeability and capillary flow	Low signal to noise ratio
**Phase-contrast MRI [62]**	Flow and pulsatility in main vessels and cerebrospinal fluid spaces	Noninvasive	Lacks resolution to assess small vessel flow
**Blood oxygen level dependent (BOLD) MRI [21]**	Cerebrovascular reactivity upon respiratory/neuronal challenge	Noninvasive; marker of brain hemodynamics and cerebrovascular reactivity	Complicated interpretation
**Non-MRI**			
**Transcranial Doppler Sonography [36]**	Cerebral blood flow and vascular reactivity in large arteries upon vasoactive stimulus	Noninvasive; quantification of vascular reactivity	Lacks resolution to assess small vessel flow
**Retinal vasculature imaging (fundus photography, optical coherence tomography [OCT] [angiography]) [41, 45]**	Retinal small vessel structure and function	Noninvasive; relatively low-cost; visualization of structure and function of vessels (at microscopic level, in the case of OCT/OCTA)	Retinal small vessels may only partly reflect cerebral small vessels
**CSF-to-serum albumin ratio [57]**	Blood-brain barrier permeability	Relatively straightforward method	Invasive

CSVD, cerebral small vessel disease; FLAIR, fluid-attenuated inversion recovery; MRI, magnetic resonance imaging; PD proton density

### Transcranial Doppler sonography

Transcranial Doppler (TCD) sonography is a technique to record the cerebral blood flow velocity and pulsatility through an artery, typically the middle cerebral artery, and thereby provides insight in the brain hemodynamics [35]. Measurements with a vasodilatory stimulus, such as breathing 6% CO_2_ in inspired air, allows assessment of vasoreactivity, a key aspect of CSVD. Although these measurements are done at the large artery level, they might also be informative of the small vessel functionality, since both are exposed to similar vascular risk factors and because large artery pulsatility may be affected by resistance of the small vessels [36]. Studies have found that pulsatility indices measured with TCD correlate with MRI derived markers of CSVD [37, 38], but further validation against other CSVD is scarce.

### Retinal vasculature imaging

Besides the brain, other organs may suffer from small vessel disease, in particular those that also receive significant portions of cardiac output, such as the kidney and retina [39]. Since the risk factors for small vessel disease in those organs are similar to CSVD, and perhaps the underlying mechanisms are as well, the vasculature in other organs may be informative for that in the brain [40]. Especially the retinal vasculature is a useful marker of CSVD, because it is also part of the central nervous system and contains close anatomical and physiological parallels with the brain vasculature [41, 42]. In contrast to the brain vasculature, the retinal vasculature is relatively easily accessible for imaging. The most commonly used modality is fundus photography which allows assessment of vessel diameters. For instance, arteriolar narrowing and venular dilation have been described as features of CSVD [41]. Higher resolution non-invasive imaging can be obtained with optical coherence tomography (OCT), and with the novel OCT angiography (OCTA). OCT and OCTA enable microscopic visualization of retinal vessels, including capillaries, and, as such, metrics like vessel wall thickness, lumen diameter, and capillary, or perfusion, density become available [40, 43]. Several recent studies found that persons with CSVD have reduced capillary density [44-46]. An important advantage of retinal vasculature imaging is that the structure and function of the vasculature can be determined more directly and at the level of the small vessels, which may allow for earlier detection [41]. A limitation remains to what extent the retinal vessels are reflective of the entire brain circulation.

## Aspects of CSVD insufficiently captured by contemporary markers

In existing literature on population-based research, CSVD is mostly reflected by lesions visible on brain MRI, as described above. While these markers have the advantage of being relatively easy to measure, there are important limitations in terms of the pathologies that can be visualized, and, more importantly, that they fail to visualize.

A first concern is the accuracy with which brain MRI is able to detect the pathologies that result from CSVD. It has become clear that MRI reveals only the tip of the iceberg of the brain injury related to CSVD and is unable to capture the complexity and heterogeneity of those lesions [47, 48]. Pathologies with a similar radiological appearance may be different in neuropathological composition, as was for instance shown in WMHs [48, 49]. Importantly, the visible lesions are merely the permanent, late-stage tissue lesions, while the majority of preceding brain injury is thought to be dynamic and possibly reversible. For example, acute tissue lesions such as small infarcts may eventually present as a WMH or lacune, or disappear [10]. Depending on the location and involvement in certain white matter tracts, even small lesions can have major consequences that are difficult to predict with conventional markers of CSVD. Increasing implementation of MRI with higher resolution or sequences that quantify microstructural integrity might enable the detection of more subtle lesions. These have, for example, already shown that pathological changes occur in the normal appearing white matter and grey matter [50, 51].

A second major limitation of most CSVD markers is that they do not directly measure functionality or pathology of the small vessels, as is possible for the cerebral large vessels. This means that mechanisms that form the basis of CSVD, namely the functionality of the neurovascular unit, or the lack thereof, remain unmeasured. There is a wide gap between CSVD-related brain pathologies as seen on MRI, and the assumed underlying processes, namely failures in meeting metabolic demands, impairment in neurovascular coupling, morphological changes of small vessel, endothelial dysfunction, blood-brain barrier leakage, cerebrospinal fluid (CSF) dynamics, inflammation and oxidative stress. Broadly speaking, the wide variety of processes that are deemed relevant in CSVD are simplified into presumed consequences of CSVD that are revealed on brain MRI. It is obvious that microscopic visualization of the small vessel functionality is extremely challenging, if not impossible, in vivo in the human brain. Nevertheless, in order to better understand the etiology and consequences of CSVD, it is essential to narrow the gap between the pathological processes and currently used markers.

While the majority of population-based studies relies on brain MRI for markers of CSVD, studies have also been conducted using TCD and retinal vasculature imaging. Those methods may overcome part of the abovementioned limitations. For example, they may show more dynamic features, including vasodilatory abilities or even compliance with metabolic demands. However, TCD lacks the spatial resolution to quantify vasoreactivity of the smaller vessels and instead measures reactivity of the large cerebral vessels that have different morphology and do not have the same regulation through neurovascular coupling. Retinal vessel imaging allows microscopic visualization of small vessels and their function, but, despite the similar origin, there are still substantial differences with the cerebral small vessels. So far, it remains largely unclear to what extent retinal vasculature features are predictive for CSVD [41].

Taken together, conventional markers for CSVD mainly detect static, end stage lesions in the brain tissue. Markers to detect subtle and dynamic injury present in earlier stages, ideally more directly related to the functionality of the small vessels, may be needed to better understand CSVD and its consequences. This will undoubtedly help identifying etiological factors and understanding the further pathophysiology. Furthermore, particularly now that assessment of other brain pathologies related to dementias becomes accessible in a more direct manner through PET-CT or CSF biomarkers (for instance, amyloid-β, tau and neurofilament light), the role of CSVD in dementia might become under-appreciated due to limited markers (Table 1).

## Alternative measures of CSVD in population-based studies

Methods for measuring CSVD in vivo in humans should ideally be able to quantify important pathophysiological processes such as endothelial dysfunction, impaired vasoreactivity, impairment of the blood-brain barrier, CSF dynamics, and waste clearance. Several techniques exist that allow quantification of those aspects, although, presently, most are not suitable for wide implementation in population-based research, due to high costs and invasive measurements.

In terms of non-invasive brain imaging, part of the gap between pathophysiology and brain imaging markers could be narrowed with more advanced brain imaging techniques [52]. Feasible methods include higher resolution MRI and assessment of white matter integrity using DTI to detect smaller brain pathologies and functional changes related to CSVD. With ultrahigh magnetic field MRI (7 Tesla or higher), luminal narrowing of small arteries can be visualized, even without contrast administration, although long scan times and high costs still preclude broad implementation [53].

Cerebral perfusion and vascular reactivity have central roles in CSVD. While cerebral perfusion is relatively easily measured as global cerebral blood flow in resting state, it is not a good indicator of the ability to match blood supply to tissue demands [21]. Novel imaging techniques enable estimation of blood flow pulsatility in small arteries, for example through 7T MRI, which is more meaningful for CSVD [54]. Perfusion MRI and blood oxygen level dependent (BOLD) MRI may also provide valuable measures of brain perfusion and subsequent oxygen extraction. For example, (capillary) transit time heterogeneity derived from perfusion MRI is thought to reflect capillary dysfunction: the transit time could either be delayed due to abnormal vessels (e.g. with luminal dilation) or shortened due to shunting from arterioles to venules (resulting in relative tissue hypoxia) [21]. These metrics are generally obtained with contrast-based imaging, but non-invasive methods such as arterial spin labelling and phase-contrast MRI are promising alternatives [55].

Quantification of blood-brain barrier leakage can be achieved through imaging, predominantly dynamic contrast-enhanced MRI [56], or through non-imaging methods, such as the CSF-to-serum albumin ratio [57]. Less invasive alternatives, such as the permeability surface-area product based on the permeability of the blood-brain-barrier to water, have also been described, but not widely used [58, 59]. Details of the abovementioned modalities can be found in the table and in the accompanying references.

Since small vessel disease likely is a systemic condition, the vasculature of other organs with similar vasculature, including the retina as described above, may be informative of CSVD. However, it remains largely unclear to what extent vascular pathologies detected in organs other than the brain align with those inside of the brain, because associations have mainly been determined with end-stage CSVD lesions in the brain. Besides imaging of the retinal vasculature, non-invasive techniques to visualize the microcirculation in other tissues may also prove valuable. An example of such imaging methods is sidestream dark field imaging, which allows visual assessment of microvessel structure, flow and functionality, and is currently mainly applied sublingually [60].

## Conclusions and directions

At present, the pathogenesis of CSVD remains incompletely understood. In addition, there is a remarkable degree of variation in the nature and severity of clinical symptoms associated with CSVD that cannot be explained fully by currently accepted markers. It is plausible that this is in part due to shortcomings in the assessment of small vessel functionality and in vivo detection of pathologies in humans. Brain MRI is the most commonly used method for CSVD detection, but relies on the visualization of hemorrhagic and ischemic brain parenchymal lesions that are sequelae of CSVD and not per se CSVD itself. Yet, these markers have almost become the definition of CSVD, while the pathologies that form the basis of CSVD, including impairments of vessel walls and the blood-brain barrier and disturbed compliance of perfusion with metabolic demands, are not detected. In order to unravel mechanisms through which CSVD might lead to cognitive deficits, the implementation of techniques that more closely reflect small vessel functionality in in vivo human studies is a priority. Research in a population-based setting will be valuable in capturing the early stages of CSVD, that might still be non-symptomatic and reversible, and to enable studying the contributions of small vessel functioning over time and relative to other brain pathologies. To that end, innovative techniques are crucial that are minimally invasive and independent to implement, while at the same time allow direct visualization and quantification of small vessel functionality, for example through reactivity.
